# Prevalence of functional gastrointestinal disorders in infants and young children in China

**DOI:** 10.1186/s12887-021-02610-6

**Published:** 2021-03-17

**Authors:** Ying Huang, Serene Yaling Tan, Panam Parikh, Vanitha Buthmanaban, Shaman Rajindrajith, Marc Alexander Benninga

**Affiliations:** 1 Department of Gastroenterology, Children’s Hospital of Fudan University, Shanghai, P.R. China; 2 FrieslandCampina, Amersfoort, The Netherlands; 3 FrieslandCampina Development Centre AMEA, 89 Science Park Drive, #02-05/06/08 The Rutherford B, Science Park 1, Singapore, 118261 Singapore; 4 Department of Pediatrics, Faculty of Medicine, University of Colombo, Colombo, Sri Lanka; 5 Department of Pediatric Gastroenterology, Emma Children’s Hospital, Amsterdam UMC, Academic Medical Center, Amsterdam, 1105 AZ The Netherlands; 6 Academic Medical Center, Amsterdam, 1105 AZ The Netherlands

**Keywords:** Colic, Regurgitation, Constipation, Diarrhea, Toddler

## Abstract

**Background:**

The prevalence of functional gastrointestinal disorders (FGIDs) in children, particularly from Asia, is largely unknown. There are not many studies done in Asia especially using the Rome IV criteria. The aim of this study is to assess the prevalence of FGIDs in infants and young children in a representative sample in China.

**Methods:**

A prospective, cross-sectional, community-based survey was conducted among healthy infants and young children between the ages of 0–4 years in Jinhua and Shanghai, China. A total of 2604 subjects (1300 subjects from Jinhua and 1304 subjects from Shanghai) completed a validated questionnaire on pediatric gastrointestinal symptoms. FGIDs in infants and young children were diagnosed using the Rome IV criteria.

**Results:**

According to the Rome IV criteria, the prevalence of having a FGID in Chinese infants and young children is 27.3%. Infant regurgitation (33.9%) was the most common FGID among the 0–6 months old while functional constipation (7.0%) was the most common among the 1–4 years old. Risk factor analysis revealed that prevalence of infantile colic was higher with better maternal education and low birth weight. Prevalence of infantile regurgitation was significantly greater in males, living in a rural area, being exclusively breast fed at least up to 4 months and starting formula feeds within the first month. The risk of functional constipation was lower for infants who were delivered vaginally.

**Conclusions:**

Infantile regurgitation was the most common FGID in Chinese infants while functional constipation was most prevalent among young Chinese children.

**Trial registration:**

Netherlands Trial Registry Identifier: NL6973/NTR7161.

**Supplementary Information:**

The online version contains supplementary material available at 10.1186/s12887-021-02610-6.

## Background

Functional Gastrointestinal Disorders (FGIDs) in infants and toddlers are a group of disorders with a variable combination of often age-dependent, chronic, recurrent symptoms in the absence of recognized organic pathology [1]. The etiology of these disorders remains uncertain and thought to be due to subtle alteration of the normal development or maladaptive behavioral response to a variety of internal or external stimuli [1]. FGIDs are observed in infants and young children worldwide with a variable frequency [2]. According to the Rome IV criteria introduced in 2016, there are seven clinical entities of FGIDs in this age group [1].

As a group of disorders, FGIDs in young children are a major challenge to the already overstretched pediatric gastroenterology services across the world. An estimated 27–40.5% infants and toddlers were reported to meet the criteria for at least one FGID [3–6]. Most of these disorders are treated with a variety of ineffective therapeutic modalities which also add a significant expenditure to healthcare systems in resource poor countries [7]. In addition, FGIDs in infancy are linked with detrimental repercussions such as maternal depression, poor infant and mother bonding, infantile abuse and poor physical growth [8, 9]. Infants and young children with FGIDs visit medical professionals more frequently and experience poor quality of life [4, 10], and in early infancy can lead to needless suffering of the caregivers [1].

Asia, home to over 50% of the world’s childhood population [11], and is going through a rapid economic transition and cultural globalization. It is known that the prevalence of FGIDs may vary with cultures, race, ethnic differences and geographic areas [12]. The current study aims to evaluate epidemiological patterns and risk factors of FGIDs in infants and young children in China using Rome IV criteria.

## Methods

### Study design and geographic details

We performed a prospective, cross-sectional, multicenter study to assess the community prevalence of FGIDs in Chinese infants and young children, aged 0–4 years. Recruitment was conducted in well baby clinics located in 3 community hospitals in Jinhua and 1 community hospital in Shanghai. These community hospitals are government hospitals where local infants and young children visit for routine medical checks and/or vaccination. Shanghai and Jinhua cities were selected as study sites to ensure a socioeconomically diverse sampling. Shanghai, a global financial hub is a tier 1 city in China, while Jinhua is a tier 2 city. Tier 1 cities are the largest and wealthiest cities with GDP over US$300 billion and a population over 15 million people. As the tiers progress, the cities decrease in size, affluence and population. Nearly 90% of the population in Shanghai lives in the urban areas compared to only 68% in Jinhua.

### Informed consent and data collection

Data were collected from May 2018 to November 2018. Infants and toddlers were recruited if consent was provided and in the absence of any organic disease. Written informed consent was obtained from the mother/caregiver after providing details of the survey and all concerns answered, before the questionnaire was introduced.

A previously validated questionnaire designed to diagnose FGIDs in infants and young children was administered to the mothers for data collection [13–15]. The questionnaire was translated into local language by a professional translation company and tested for clarity and applicability using a smaller sample of mothers of infants and young children. The final version of the questionnaire (in English language) is provided as an additional file (see Supplementary file 1). The questionnaire had 3 sections: Section 1 contained questions on demographics characteristics including age, gender, birth order, weight, height, growth pattern, family size as well as feeding practices; Section 2 contained explicit questions on gastrointestinal symptoms including stool pattern, spitting and crying habits for infants and young children; Section 3 collected information on the family social and economic details such as place of residence as well as parental education level and employment status. The last section also included questions on adverse life events faced by the child and family, such as exposure of child or mother to physical or verbal abuse. The Rome IV criteria were used to diagnose FGIDs [1].

### Sample size calculation

The sample size was calculated using an estimated 10% prevalence for infant colic and childhood constipation [3]. Assuming a confidence level of 95%, power of 80% and a precision found to the nearest 2%, a sample size of 1300 infants in ages 0–6 months (group 1) and 1300 young children in ages 7 months – 4 years (group 2) were considered adequate. Group 2 was further stratified for age according to the following categories; 7–12 months, 1–2 years, 2–3 years and 3–4 years. For each group, equal number of infants and toddlers were recruited from Shanghai and Jinhua.

### Statistical analysis

The data from both sites (Shanghai and Jinhua) were entered into a Microsoft Excel (Microsoft Office 365 MSO, 16.0.10730.20438). We used SAS software (Version 9.4, SAS Institute Inc., Cary, NC, USA) for the data analysis. Data from both sites were pooled for analysis. Descriptive statistics was used to present the characteristics of the sample and the prevalence rates FGIDs. Multivariate logistic regression was used to evaluate the potential risk factors (such as demographic data, exposure to adverse events, social and economic factors) associated with the development of FGIDs. Odds ratio (OR) estimates and 95% confidence interval of OR, as well as the *p*-values of the Wald chi-square test for each risk factor were provided. All statistical tests used a significance level of 0.05. Multiple logistic regression was performed on variables that were found to have significant associations.

### Ethical approval

The protocol, information letter to the parents/caregivers and written informed consent form were approved by the Research Ethics Board of Children’s Hospital of Fudan University, Shanghai, China. The survey was conducted in accordance with the guidelines of the Declaration of Helsinki and the International Conference on Harmonization (ICH) guidelines on Good Clinical Practice (GCP). The study was registered in the Netherlands Trial Registry (identifier: NL6973/NTR7161).

## Results

### Sample characteristics

Mothers of 3070 infants and young children between the age of 0–4 years old were invited to participate in the study. Of these, 2791 mothers (1393 from Jinhua and 1398 from Shanghai) filled in the questionnaires. Completely filled 2604 questionnaires (1300 from Jinhua and 1304 from Shanghai) were included in the final analysis. Data was excluded from subjects who refused to sign the consent form (*n* = 140), did not complete the screening procedure (*n* = 139) and did not complete the questionnaire (*n* = 187).

All subjects recruited were Chinese, with a mean age of 14 months ±14.1 SD (median 7 months; age 0–48 months) and 1329 (51%) were males. Most subjects were living in the urban areas (61%) and 43% of the families had an annual household income between US$20,830 – US$36,450. In terms of parental educational background, the majority of mothers (48.8%) and fathers (51.0%) had a Bachelor’s degree.

### Prevalence of FGIDs in infants and young children

In total, 712 subjects (27.3%) were diagnosed with at least one FGID, according to the Rome IV criteria. Ninety-seven infants (3.72%) had more than one FGID of which the most common combination was infant colic and regurgitation in 71 infants (2.73%). The prevalence of FGIDs among infants and young children according to residence area and age group is presented in Table 1. Infant regurgitation was the most prevalent FGID in early infancy (33.9%) and it was higher among babies dwelling in rural areas (31.2% versus 25.2%, *p* = 0.014). Functional constipation was the most common disorder in the 7 months – 4 years old’s (7.0%).

**Table 1:** Prevalence of self-reported FGIDs according to residence area and age group

FGIDs	Age Groups	%	Rural (Total: ***n*** = 1008)	Urban (Total: ***n*** = 1596)	***p***-value
Infant Colic	0–6 months	14.9%	14.7%	15.1%	0.991
Infant Regurgitation	0–6 months 7–12 months	33.9% 3.4%	31.2%	25.2%	0.014*
Infant Dyschezia	0–6 months 7–9 months	3.6% 2.7%	3.4%	3.7%	0.480
Functional Diarrhea	7–12 months 1–4 years	2.2% 2.7%	2.8%	2.4%	0.324
Functional Constipation	0–6 months 7–12 months 1–4 years	1.5% 1.5% 7.0%	4.6%	2.9%	0.196

*P = < 0.05

A total of 43.8% of subjects developed colic between the 1st – 2nd month, and 30.9% of infants were noted to have regurgitation between 2nd – 3rd month. There were two peak onsets for constipation: 4th – 5th month in early infancy (31.6%) and between 23rd – 30th month for subjects aged 7 months – 4 years (26%).

### Association between FGIDs and early life factors and infant feeding practices

Table 2 shows the association between FGIDs and early life factors and infant feeding practices [see Additional file 2]. The risk of having infant colic was significantly higher among infants with low birth weight (2.1–2.5 kg) (OR = 7.92, 95%CI = 1.37–45.85, *p* = 0.021). Being a male (OR = 1.38, 95%CI = 1.04–1.82, *p* = 0.024), exclusively breastfed between 1 and 4 months [(1–2 months: OR = 4.54, 95%CI = 2.33–8.84, p = <.0001), (2–3 months: OR = 9.27, 95%CI = 4.71–18.26, p = <.0001), (3–4 months: OR = 4.29, 95%CI = 2.43–7.58, p = <.0001)] and starting formula between 0 and 1 month (OR = 2.42, 95%CI = 1.46–3.99, *p* = 0.001) increased the risk of infant regurgitation. The risk of functional constipation was lower if the child was delivered vaginally (OR = 0.005, 95%CI = < 0.001–0.17, *p* = 0.003).

After multiple logistic regression analysis, breast feeding for 2 months or more (adjusted OR = 1.79, adjusted 95%CI = 1.36–2.37, adjusted p = <.0001) was significantly associated with infant regurgitation; birth weight of 2.5 kg or less (adjusted OR = 3.47, adjusted 95%CI = 1.01–11.89, adjusted p = < 0.048) was significantly associated with infant colic.

### Association between FGIDs and socio-demographic characteristics

Table 3 reflects the association between FGIDs and socio-demographic factors [see Additional file 3]. The analysis revealed that staying in the rural area significantly increased the risk of infant regurgitation (OR = 1.44, 95%CI = 1.08–1.93, *p* = 0.014). Higher maternal education level (Master’s degree) significantly increased the risk of infant colic (OR = 3.39, 95%CI = 1.02–11.33, *p* = 0.047) and functional diarrhea (OR = 8.93, 95%CI = 1.07–74.55, *p* = 0.043).

### Association between FGIDs and stressful life events

Table 4 shows the association between FGIDs and prenatal and postnatal stressful events faced by the mother and the child [see Additional file 4]. The majority reported that neither they nor their children were exposed to any of the listed stressful events during the period. No significant relationship was found between these stressors and FGIDs (*p* > 0.05).

## Discussion

To the best of our knowledge, this is the first paper that reports epidemiology and risk factors of FGIDs in infants and young children in Asia using the Rome IV criteria. We observed 27.3% of infants and young children having at least one FGID and 3.72% infants having more than one FGID, of which colic and regurgitation (2.73%) was the most find combination. Infantile regurgitation was the most common FGID among Chinese infants while functional constipation was the most prevalent FGID in Chinese toddlers.

In accordance with our study, two recent studies that used Rome IV criteria [7, 10] and a systematic review that used Rome II and III criteria [3] reported at least 30% of infants and young children suffering from at least one FGID. This is in line with the prevalence seen in our study among Chinese infants and young children. An earlier Chinese study, conducted in Shanghai, which used the Rome III criteria noted a slightly higher prevalence of 38% in subjects aged between 6 and 24 months [16].

Infantile regurgitation has been reported as the most common FGID across the world in infants and young children [5–7, 10, 17]. The current study also found a nearly 34% prevalence of infant regurgitation in the 0–6 months age group which decreased to less than 5% at the end of the first year. Comparing our results with the African (40%), American (24%) and European (14%) studies which also used the Rome IV criteria, prevalence of infantile regurgitation in the current study was closer to the African study [7, 10, 18]. This difference in prevalence could be due to the small sample size (*n* = 58) of infants in the study conducted in America. The low prevalence in the European study could be due to the fact that the subject’s parents were interviewed by a healthcare professional, suggesting different interpretation of symptoms.

Our study found that being male, living in a rural area and consumption of formula feeds between 0 and 1 month increased the risk of infant regurgitation while being exclusively breastfed for 4–6 months reduced the risk by 99% (OR = 0.01, 95%CI = < 0.001–0.09, p = < 0.001). In contrast, the majority of the studies did not find a significant difference in the prevalence of FGIDs between genders [5, 10, 19], whereas according to our data, males had 38% higher risk of infant regurgitation (OR = 1.38, 95%CI = 1.04–1.82, *p* = 0.024). The pathophysiology of regurgitation includes small esophageal volume combined with immaturity of lower esophageal sphincter, overfeeding and infant positioning [1]. Parents in general tend to overfeed while bottle feeding, as they are less likely to respond to child’s satiety cues [20]. This is further exacerbated by Chinese cultural practice of feeding higher volumes of milk (>840mls/day) to the male infants at 3 months of age compared to female infants [21] and the belief that a fat baby is a healthy baby [22]. This may explain the higher predisposition for infant regurgitation seen in males. Furthermore, our study shows that infants staying in the rural areas had a 44% increased risk of developing infant regurgitation (OR = 1.44, 95%CI = 1.08–1.93, *p* = 0.014), which may be due to parental unawareness of the correct feeding position and more persistent beliefs in the traditional health image, secondary to the differences in access to care and provider practices [23–25].

Infants who are being breastfed have the ability to self-regulate milk consumption resulting in different feeding patterns of increased frequency and decreased volumes, which is a factor associated with reduced reflux [26]. Additionally, breast fed infants have more rapid gastric emptying, therefore a lower esophageal pH which is more likely to stimulate peristalsis and thus shorter the duration of reflux [27]. Interestingly, our study also found that infants who initiated formula feeding between 3 and 4 months old were 80% less likely to have regurgitation. This is in contradiction to an earlier study in Italy which reported no difference in infant regurgitation based on type of feeding (breast-fed, mixed-fed and bottle-fed) [28]. A plausible explanation could be that the peak age for regurgitation in our study is between the 2nd – 3rd month and thus, the prevalence rate can be expected to drop after the 3rd month.

The prevalence of infant colic (14.9%) in the 0–6 months age group in our study is much higher than that reported in studies in the US (5.9%) [5] and China (1.4%) [16]. The difference in the prevalence can be attributed to the use of Rome IV criteria in our study, which replaced the strict “rule of threes” with a diagnostic criteria of “recurrent and prolonged periods of crying, fussing or irritability” [1]. It is expected that this change will result in increased diagnosis and thereby, higher reports of colic in future studies using the Rome IV diagnostic criteria. This can be seen in the most recent study in Africa, where infantile colic was reported to be 57.6% [7].

In line with two Danish studies, our results indicate that having a low birth weight (2.1–2.5 kg) was associated with an increased risk of infant colic [29, 30]. Søndergaard et al. reported that low birth weight infants (< 2.5 kg) had more than twice the risk of having infant colic [29]. The second study reported increasing risk for infantile colic with decreasing gestational age [30]. Furthermore, small for gestational age infants with birthweight below 10th percentile had increased odds of infant colic (OR = 1.2, 95%CI = 1.1–1.3) [30]. It has been reported that mothers tend to pressure feed infants with lower birth weight by manipulating the frequency and/or quantity of milk feeds [31] leading to potential overfeeding. Due to the immaturity of the infant gut [32], the infant may not be able to handle the large volume of milk feeds, thereby leading to infant colic.

The prevalence of functional diarrhea reported in this study was low in both the 7–12 months (2.2%) as well as in the 1–4 years (2.7%) age group. Tilburg et al. reported a similar prevalence for infants < 1 year (2.4%) using Rome III criteria, but the prevalence in young children (1–3 years) was much higher at 6.4% [5]. More recently, another study using Rome IV criteria found no occurrence of functional diarrhea in infants or toddlers [10]. This difference in prevalence could be due to increase in defecation frequency from 3 to 4 stools per day and elimination of the criterion of passing of stools during sleep in the Rome IV criteria [33]. These changes in criteria may explain the reduction of prevalence in functional diarrhea when using the Rome IV criteria seen in our study and reported in the US study [10].

The prevalence rate for functional constipation was 1.5% (0–6 months age group) versus 7% (1–4 years age group) in our study. These data concur with previous studies, that report a higher prevalence of functional constipation in young children and a median age of onset of 27.6 months [5, 6, 34]. Our study showed that vaginal birth was significantly associated with lesser risk of functional constipation. However, a cohort study (*n* = 83,019) conducted by the Japan Environment and Children’s Study concluded that there were no significant differences in the risk ratio for functional constipation with cesarean section and vaginal birth [35]. Cesarean delivery has been associated with various infantile conditions such as inflammatory bowel disease and gastroenteritis [36, 37]. It also disturbs the normal colonization of the infant gut by preventing exposure to mothers’ microbes [38]. On the other hand, infants born via vaginal delivery are exposed to the mother’s vaginal and intestinal microbiota which is important in the development of the infant’s immune system [35, 39, 40] and may reduce the development of functional constipation [41]. In contrast to the recent systematic review and meta-analysis [42], our study did not find any association between functional constipation and geographical location, lifestyle factors as well as stressful life events.

Stressful life events are known to predispose children to develop FGIDs. Several studies among older children have reported higher risk for functional constipation and functional abdominal pain disorders with stressful life events related to home and school [43, 44]. However, a study conducted in preschool Palestinian children reported otherwise [45]. In addition, exposure to child maltreatment is also known to predispose children to FGIDs [43, 46]. A study conducted among children of 6–48 months in Sri Lanka reported higher prevalence of functional constipation in children subjected to violence and living with mothers exposed to violence [13]. We therefore, hypothesized maternal exposure to violence and adverse behavior towards the baby may increase the risk of FGIDs in infants and young children. However, in our study, the majority of the study population reported good to excellent domestic harmony and no exposure to violence. We also did not find any association between FGID and growth faltering as has been reported in a previous study from Shanghai [16] and Sri Lanka [13].

We also acknowledge that there are few limitations in this study. Although we recruited a large sample size from both rural and urban areas of China, we used parent reporting to interpret child’s symptoms. This reporting may be inaccurate as infants and younger children are not be able to verbalize their symptoms. In addition, parents who have children with symptoms are more prone to response as well. On the other hand, parents may not be aware of non-observable symptoms [10] and child’s bowel symptoms [47] in older children especially if the infant or young children is being sent to the day care.

## Conclusions

In conclusion, we found that 27.3% of infant and young children fulfilled the criteria for at least one FGID. FGIDs are common in infants and young children living in China. Our data indicate that the most common FGIDs in infants were regurgitation and colic, while functional constipation was most prominent FGID among young children. Factors such as gender, maternal education, residence area, mode of delivery, duration of exclusive breastfeeding and age of formula initiation showed a significant association with different FGIDs. Longitudinal studies are needed to assess diagnostic stability and the impact of FGIDs throughout childhood and into adulthood.

## Supplementary Information

**Additional file 1: Supplementary file 1.** Questionnaire for assessment of Functional Gastrointestinal Disorders in infants and young children.

**Additional file 2: Table 2.** Early life factors and infant feeding practices against FGIDs. Associations between early life factors and infant feeding practices against FGIDs.

**Additional file 3: Table 3.**Socio-demographiccharacteristics against FGIDs. Association between socio-demographiccharacteristics against FGIDs.

**Additional file 4: Table 4.** Stressful life events against FGIDs. Association between stressful life events against FGIDs.

## Data Availability

The datasets used and/or analyzed during the current study are available from the corresponding author on reasonable request.

## Abbreviation

FGIDs: Functional Gastrointestinal Disorders
