# Supplementary material for: Prevalence of functional gastrointestinal disorders in infants and young children in China
Source: BMC Pediatr. 2021 Mar 17;21:131. doi: 10.1186/s12887-021-02610-6 (PMC7968152; doi:10.1186/s12887-021-02610-6)
Supplement: Supplementary file 1 — Additional file 1: Supplementary file 1. Questionnaire for assessment of Functional Gastrointestinal Disorders in infants and young children. [file 12887_2021_2610_MOESM1_ESM.doc]

**Annex 1: Questionnaire for infants aged 0 – 6 months**

**Assessment of Functional Gastrointestinal Disorders**

**Among Infants Aged 0 to 6 months**

Date:....................................Child Subject Number...................................

Health Center……………………………………...................................................

Location: ___ ……, ____ ……, ____ ……..

Questionnaire completed by:………………………………………………………………………………………………………

Part 1 and 2 of the questionnaire will be completed by interview of the parent.

Part 3 of the questionnaire is self-administered and will be completed by the parent.

**PART 1**

**Information about the child:**

1. Initials: .....................................................................................
2. Date of Birth: ............................................ Age (mo) ..........................
3. Sex: ___ male, ___ female
4. How many siblings: number of females………….number of males..............
5. What is the birth order? .............................................................
6. Gestational age: ……………………………… weeks
7. Prenatal complications? ………Yes …………No
8. If yes, please specify ……………………………………………………………………
9. Mode of delivery ….Vaginal ….Elective C-section….Emergency C-section…Forceps delivery

Reason for non-vaginal delivery ……………………………...

1. Birth weight: ……………………………... grams
2. Current weight: ……………………………… grams
3. Current height : ………………………………. cm
4. Admission to special care baby units/neonatal interventions after delivery? ………Yes …………No

If yes, please specify ………………………………………………………………………………………………

1. Antenal complications ………Yes …………No

If yes, please indicate ……Diabetes Melliltus/Gestational Diabetes

……Hypertension ……bleeding……Heart disease………IUGR………others

1. Initiation of breast feeding ………Yes …………No

If yes, please specify at what age and duration and if exclusive…………………

1. Initiation of formula feeding ………Yes …………No

If yes, please specify at what age and duration……………………………………………………………

1. Current type of feeding (more answers are possible): ___ breastfeeding,

___ formula feeding, ___ additional foods

1. a. Growth curve (see Child Health Record)? ____ normal ___ abnormal

b. If abnormal, which growth curve (see Child Health Record)?

- 1. ___ overweight (> 2SD weight for length)
  2. ___ underweight (< 2SD weight for age)
  3. ___ stunting (< 2 SD length for age)
  4. ___ wasted (< 2SD weight for length)

1. Number of health care consultations due to symptoms other than abdominal pain

| Number of health care consultations during last 6 months | Family doctor | pediatrician | Hospital | Main symptom | Result | 1.Better than previous  2.No change  3.Worse than previous |
| --- | --- | --- | --- | --- | --- | --- |
|  |  |  |  |  |  |  |
|  |  |  |  |  |  |  |
|  |  |  |  |  |  |  |
|  |  |  |  |  |  |  |

1. General medical history, including gastrointestinal disorders diagnosed: ………………………………………………………………………………

**PART 2**

**This section asks about your baby’s defecation pattern, spitting up and excessive crying.**

1. In the last 2 weeks, how often did your baby usually have poop?
   1. __ 2 times a week or less
   2. __ 3 to 6 times a week
   3. __ Once a day
   4. __ 2 to 3 times a day
   5. __ More than 3 times a day
2. In the last 2 weeks, what was your baby’s poop usually like (show picture)?
   1. __ Type a: Watery
   2. __ Type b: Soft
   3. __ Type c: Formed
   4. __ Type d: Hard
   5. __ It depends (his/her poops are not always the same)
3. In the last 2 weeks, did your baby have pain during bowel movement?
   1. __ Never
   2. __ Once in 2 weeks
   3. __ Once a week
   4. __ More than once a week
   5. __ Once a day
   6. __ More than once a day
4. In the last 2 weeks, how often did your baby pass blood with stools?
   1. __ Never
   2. __ Once in 2 weeks
   3. __ Once a week
   4. __ More than once a week
   5. __ Once a day
   6. __ More than once a day
5. In the last 2 weeks, did your baby have to strain or cry before (un)successful passage of soft stools?
   1. __ Never
   2. __ Once in 2 weeks
   3. __ Once a week
   4. __ More than once a week
   5. __ Once a day
   6. __ More than once a day
6. When your baby strains or cries, does it last for 10 minutes or more ?
    1. __ No
    2. __ Yes

27. In your opinion, is the stool pattern a problem for your baby?

- 1. __ No
  2. __ Yes

28. Except for difficult pooping, is your child healthy?

1. __ No
2. __Yes

29.Did you ever visit a doctor because your baby had stool problems?

1. __ No*🡪 continue with question 32*

2. __ Yes

30.a. Did a doctor or nurse ever examine your baby and say that your baby had a huge poop inside?

1. __ No

2. __ Yes

b. If yes, at what age? ……………………. months

c. If yes, what tests did the doctor do (more answers are possible)?

- 1. __ Examination abdomen
  2. __ Anal and rectal exam
  3. __ Blood test: ……………………………………
  4. __ X-ray: ……………………………………
  5. __ Ultrasound: ……………………………………
  6. __ Other: ……………………………………
  7. __ No test

31.a. Has your baby ever been treated for constipation by a doctor?

1. __ No

2. __ No, self-treatment or pharmacy advise

3. __ Yes

b. The treatment included (more answers are possible):

1. Dietary advice:

1. ___ switch from breastfeed to formula feed
2. ___ use other type of formula

2. Oral laxatives:

1. ___ tablet form ……………………………………….
2. ___ syrup form …………………………………………
3. ___ powder form ………………………………………

3. Rectal laxatives:

1. ___ glycerin supp
2. ___ other supp……………………………………………………..
3. ___ rectal enema
4. ___ soap
5. ___ stimulation with thermometer
6. ___ other ……………………….………………………..

4. __ Herbal remedies, please specify…………………………………………………….

5. __ Other treatment, please specify……………………………………………………

32. In the last 2 weeks, how much did your baby spit or vomit?

1. __ Never

2. __ Once in 2 weeks

3. __ Once a week

4. __ More than once a week

5. __ Once a day

6. __ 2 to 5 times a day

7. __ More than 5 times per day

33. . If your baby regurgitates (spits up food) more than 2 times a day, did this happen for at least 3 weeks?

1. __ No

2. __ Yes

34. . Has one of the following ever occurred?

1. ___ Retching

2. ___ Hematemesis

3. ___ Wheezing or trouble breathing

4. ___ Aspiration

5. ___ Apnea

6. ___ Failure to thrive

7. ___ Feeding difficulties

8. ___ Swallowing difficulties

9. ___ Abnormal posturing

10. __ None of the above

35. . Is your child healthy, except for spitting up or vomiting?

1. __ No

2. __ Yes

36. . For the past 3 months *when not vomiting* did your child bring up food in his/her mouth after it has already been chewed and swallowed?

1. __ No *🡪 continue with question 42*

2. __ Yes

37. At what age did the problem start?

1.___ Birth

2. ___ 1 - 2 months

3. ___ 3 - 6 months

38. Does the problem occur when your child is asleep?

1. __ No

2. __ Yes

39. Does the problem occur when your child is interacting with family or friends?

1. __ No

2. __ Yes

40. Does your child seem distressed during spitting up?

1. __ No

2. __ Yes

41. Is the problem better with:

a. Formula changes? __ No __Yes

b. Medicines? __ No __Yes

42. Has your child experienced at least 2 periods of unremitting paroxysmal vomiting with or without retching lasting hours to days within a six month period?

1. __ No 🡪 *continue with question 44*

2. __ Yes

43. a. Between episodes of vomiting does your child return to his/her usual state of health for weeks or months?

1. __ No

2. __ Yes

b. Are episodes separated by weeks to months?

1. __ No

2. __ Yes

44 a. In the last 2 weeks did your baby show irritability, fussing, or crying (also described as colic) that starts and stops without obvious cause?

1. __ No

2. __ Yes

b. If yes, was the average duration of all episodes 3 or more hours per day?

1. __No

2. __Yes

c. If yes, was the frequency 3 or more days per week?

1. __No

2. __Yes

d. If yes, did your baby show any signs of fever or illness?

1. __No

2. __Yes

e. If yes, at what age did these symptoms start and when did they stop?

Started at ……….months, stopped at …….. months (if applicable)

45. a. Did you ever visit this clinic because of excessive crying?

1. __ No

2. __ No, we went to another clinic or hospital

3. __ Yes

1. If so, at what age? ………… months
2. If so, what tests did the doctor do (more answers are possible)?
3. __ Physical examination
4. __ Blood test: ……………………………………
5. __ X-ray: ……………………………………
6. __ Others: …………………………………… (please specify)
7. __ No test

46. a. Has your baby ever been treated for excessive crying by a doctor?

1. __ No *🡪 continue with question 47*

2. __ No, self-treatment or pharmacy advise *🡪 continue with question 46*

3. __ Yes

b. The treatment included (more answers are possible)?

- 1. __ Exclusive breastfeeding
  2. __ Switch to formula feeding
  3. __ Hypoallergenic formula
  4. __ Soy based formula
  5. __ Hypoallergenic diet for mother
  6. __ More frequent feeding
  7. __ Less frequent feeding
  8. __ Keep the infant in upright position
  9. __ Keep the infant in side position
  10. __ More rhythm in daily routine (feeding, cleaning, sleeping)
  11. __ Carrying the baby in sling
  12. __ Medication:

___ anti-spasmolytics …………………………………

___ other ……………………………………………………

- 1. __ Herbal remedies, please specify………………………………………..
  2. __ Other:……………………………………………………………………………….

**PART 3**

**Information about the family**

47. Mother’s age in years : ......................................................................

48. Where is your family originally from? (e.g. The Netherlands, Indonesia, Turkey, Italy, Israel, Suriname, etc.): ……………………………………………………………………

49. Mother’s years of education: …………………………………………………………………..

50. Mother's employment: ......................................................................

51. Mother’s marital age in years:......................................................................

52. Marital status:

1. Married …… a. living with partner ……

b. living separate from partner ……

c. widow ……

d. divorced ……

1. Not married …… a. living with partner ……

b. living separate from partner ……

c. single parent ……

53. Father’s age in years: ......................................................................

54. Where is your family originally from? (e.g. The Netherlands, Indonesia, Turkey, Italy, Israel, Suriname, etc.): ……………………………………………………………………

55. Father’s years of education: …………………………………………………………………..

56. Father's employment: ......................................................................

57. Father’s marital age in years: ......................................................................

58. Marital status:

- - 1. Married …… a. living with partner ……

b. living separate from partner ……

c. widower ……

d. divorced ……

- - 1. Not married …… a. living with partner ……

b. living separate from partner ……

c. single parent ……

59. Household income/year:

1. ___ Less than ￥73,500,-
2. ___ ￥73,500- ￥146,999,-
3. ___ ￥147,000-￥257,249,-
4. ___ ￥257,250-￥367,499,-
5. ___ ￥367,500-￥735,000,-
6. ___ Over ￥735,000,-

60. Does your income meet your essential needs?

1. ___ No

2. ___ Yes

61. Does your family have any loans (mortgage not included)?

1. ___ No

2. ___ Yes

62. How is the relationship with your partner and family members who are living in your house?

1. ___ Very good

2. ___ Good

3. ___ Bad

4. ___ Very bad

63. Place of residence:

1. ___ Urban area

2. ___ Rural area

64. a. Was your family forced to change their place of residence because of security reasons or other related reasons?

1. ___ No

2. ___ Yes

b. If yes, in which year? …………….

65. In the last two months, have you been subject to verbal violence in your own house?

- 1. __ Never
  2. __ Once in two months
  3. __ Once a month
  4. __ More than once a month
  5. __ Once a week
  6. __ More than once a week
  7. __ Daily

66. In the last two months, have you been subject to physical violence in your own house?

1. __ Never
2. __ Once in two months
3. __ Once a month
4. __ More than once a month
5. __ Once a week
6. __ More than once a week
7. __ Daily

67. In the last two months, has your baby been subject to verbal violence in your own house?

- 1. __ Never
  2. __ Once in two months
  3. __ Once a month
  4. __ More than once a month
  5. __ Once a week
  6. __ More than once a week
  7. __ Daily

68. In the last two months, has your baby been subject to physical violence in your own house?

1. __ Never

2. __ Once in two months

3. __ Once a month

4. __ More than once a month

5. __ Once a week

6. __ More than once a week

7. __ Daily

69. Does anybody of the family is suffering from a long-lasting abdominal pain

(more than 2 months)

a. 1. ___ No

2. ___ Yes

b. If yes, what is the relationship of this family member to the subject? …………….

**Annex 2: Questionnaire for infants and toddlers aged 7 months – 4 years**

**Assessment of Functional Gastrointestinal Disorders**

**Among Children Aged 7 months to 4 years**

Date:……………………………..............Child Subject Number ................................

HealthCenter......................................................................................................................

Location: ___ …, ____ ….., ____ ……

Age category: ___ 7-12 mo, ___ 13-24 mo, ___25-36 mo, ___ 37-48 mo

Questionnaire completed by:………………………………………………………………………………………………………

Part 1 and 2 of the questionnaire will be completed by interview of the parent.

Part 3 of the questionnaire is self-administered and will be completed by the parent.

**PART 1**

**Information about the child:**

1. Initials: .....................................................................................
2. Date of Birth: .............................................. Age (mo) .......................
3. Sex: ___ male, ___ female
4. How many siblings: number of females ………….number of males..............
5. What is the birth order? .............................................................
6. Gestational age: ……………………………… weeks
7. Prenatal complications? ………Yes …………No
8. If yes, please specify ………………………………………………………………………………………………Mode of delivery….Vaginal ….Elective C-section….Emergency C-section…Forceps delivery

Reason for non-vaginal delivery

1. Birth weight: ……………………………... grams
2. Current weight: ……………………………… grams
3. Current height : ………………………………. cm
4. Admission to special care baby units/neonatal interventions after delivery? ………Yes …………No

If yes, please specify ………………………………………………………………………………………………

1. Antenal complications ………Yes …………No

If yes, please indicate ……Diabetes Melliltus/Gestational Diabetes ……Hypertension ……bleeding……Heart disease………IUGR………others

1. Initiation of breast feeding ………Yes …………No

If yes, please specify at what age and duration and if exclusive………………………………………

1. Initiation of formula feeding ………Yes …………No

If yes, please specify at what age and duration……………………………………………………………

1. Current type of feeding (more answers are possible): ___ breastfeeding,

___ formula feeding, ___ additional foods

1. a. Growth curve (see Child Health Record)? ____ normal, ___ abnormal

b. If abnormal, which growth curve (see Child Health Record)?

- 1. ___ overweight (> 2SD weight for length)
  2. ___ underweight (< 2SD weight for age)
  3. ___ stunting (< 2 SD length for age)
  4. ___ wasted (< 2SD weight for length)

1. Number of health care consultations due to symptoms other than abdominal pain

| Number of health care consultations during last 12 months | Family doctor | pediatrician | Hospital | Main symptom | Result | 1.Better than previous  2.No change  3.Worse than previous |
| --- | --- | --- | --- | --- | --- | --- |
|  |  |  |  |  |  |  |
|  |  |  |  |  |  |  |
|  |  |  |  |  |  |  |
|  |  |  |  |  |  |  |

1. General medical history , including gastrointestinal disorders diagnosed: ………………………………………………………………………………
2. Is your child mentally developing normal: __ no, __ yes

**PART 2**

**This section asks about your child’s defecation pattern and several abdominal complaints.**

1. In the last 2 months, how often did your child usually have poop?
   1. __ 2 times a week or less *🡪 continue with question 22*
   2. __ 3 to 6 times a week
   3. __ Once a day
   4. __ 2 to 3 times a day *continue with question 23*
   5. __ More than 3 times a day
2. How long has your child had bowel movements 2 times a week or less?
   1. __ Since less than 2 months
   2. __ Since 2 to 6 months
   3. __ Since 6 months to one year
   4. __ Since 1 - 2 years
   5. __ Since 2 - 3 years
   6. __ Since 3 - 4 years
3. In the last 2 months, what was your child’s poop usually like? (show picture)
   - - 1. __ Type 1: Very hard (separate hard lumps)
       2. __ Type 2: Hard
       3. __ Type 3: Dry and lumpy
       4. __ Type 4: Smooth and soft
       5. __ Type 5: Very soft blobs
       6. __ Type 6: Mushy
       7. __ Type 7: Watery
       8. __ It depends (his/her poops are not always the same)
4. In the last 2 months, did your child have pain during defecation?
   1. __ Never
   2. __ Once in 2 months
   3. __ 1 to 3 times per month
   4. __ Once a week
   5. __ More than once a week
   6. __ Every day
5. a. In the last 2 months, did your child have to strain during bowel movements?
   1. __ Never
   2. __ Once in 2 months
   3. __ 1 to 3 times per month
   4. __ Once a week
   5. __ More than once a week
   6. __ Every day

b. If so, did your child have to strain and cry at least 10 minutes before (un)successful passage of soft stools?

- 1. __ No
  2. __ Yes

1. In the last 2 months, did your child have a poop that was so big that it clogged the toilet (large diameter stool)?
2. __ No
3. __ Yes
4. Some children hold in their poop even when there is a toilet available. They may do this by stiffening their bodies or crossing their legs.
   In the last 2 months, when at home, how often did your child try to hold in a poop?
   1. __ Never
   2. __ Once in 2 months
   3. __ 1 to 3 times per month
   4. __ Once a week
   5. __ More than once a week
   6. __ Every day
5. a. Is your child toilet trained (clean) at home and outside home?
   1. __ No *🡪 continue with question 31*
   2. __ No, only at home *🡪 continue with question 31*
   3. __ Yes

b. If yes, at what age did your child become clean? ……………………………… months

1. For children who became completely toilet trained (clean):
   In the last 2 months, how often was your child’s underwear stained or soiled with poop?
   1. __ Never
   2. __ Once in 2 months
   3. __ 1 to 3 times per month
   4. __ Once a week
   5. __ More than once a week
   6. __ Every day
2. For children who became completely toilet trained (clean):
   In the last 2 months, how often was your child’s underwear soiled with urine?
   1. __ Never
   2. __ Once in 2 months
   3. __ 1 to 3 times per month
   4. __ Once a week
   5. __ More than once a week
   6. __ Every day
3. In the last 2 months, how often did your child pass blood with stools?
   1. __ Never
   2. __ Once in 2 months
   3. __ 1 to 3 times per month
   4. __ Once a week
   5. __ More than once a week
   6. __ Every day

32. In the last 2 months, did your child have any of the following symptoms?

1. __ Abdominal pain

2. __ Vomiting

3. __ Loss of appetite

4. __ Loss of weight

5. __ None of the above

33. In your opinion, is the stool pattern a problem for your child?

1. __ No

2. __ Yes

34. Did a doctor or nurse ever examine your child and say that your child had a huge poop inside?

1. __ No

2. __ Yes

35a. Did you ever visit this health center because your child had stool problems?

1. __ No *🡪 continue with question 37*

2. __ No, we went to another clinic or hospital

3. __ Yes

b. If so, at what age?...................................months

c. If so, what tests did the doctor/nurse do (more answers are possible)?

- 1. __ Examination abdomen
  2. __ Anal and rectal exam
  3. __ Blood test: ……………………………………
  4. __ X-ray: ……………………………………
  5. __ Ultrasound: ……………………………………
  6. __ Other: ……………………………………
  7. __ No test

36. a. Has your child ever been treated for constipation by a doctor?

1. __ No

2. __ No, self-treatment or pharmacy advise

3. __ Yes

b. The treatment included (more answers are possible):

1. Dietary advice:

a. __ eating more fibers

1. __ eating more fruits

c. __ drinking more water

d. __ other…………………………………………

2. Child behavior advice:

- 1. __ toilet training
  2. __ punishing
  3. __ rewarding
  4. __ other…………………………..

3. Oral laxatives:

1. ___ tablet form ……………………………………….
2. ___ syrup form …………………………………………
3. ___ powder form ………………………………………

4. Rectal laxatives:

1. ___ glycerin supp
2. ___ other supp
3. ___ rectal enema

5. __ Herbal remedies, specify……………………………………

6. __ Other……………………………………………………………

37. a. Does your child have at least 4 loose, painless stools on a daily basis?

1. __ No 🡪 *continue with question 38*

2. __ Yes

b. If so, does this last for more than 4 weeks?

1. __ No

2. __ Yes

c. How old was your child when this began?

1. __ Less than 6 months

2. __ 6 to 12 months

3. __ 1 to 2 years

4. __ 2 to 3 years

5. __ Older than 3 years

d. Does this also happen when your child is asleep?

1. __ No

2. __ Yes

e. Is your child gaining weight normally?

1. __ No

2. __ Yes

***If your child is 13 months or older, please skip to question 48***

38. a. In the past 3 months, did your child spit up or vomit at least twice per day?

1. ___ No 🡪 *continue with question 40*

2. ___ Yes

b. If yes, did this last for at least 3 weeks?

1. ___ No

2. ___ Yes

39. How many times per day does your child spit up or vomit on average?

1. ___ Twice a day

2. ___ 3-10 times a day

3. ___ More than 10 times a day

4. ___ More than 20 times a day

40. Has one of the following ever occurred?

1. ___ Retching

2. ___ Hematemesis

3. ___ Wheezing or trouble breathing

4. ___ Aspiration

5. ___ Apnea

6. ___ Failure to thrive

7. ___ Feeding difficulties

8. ___ Swallowing difficulties

9. ___ Abnormal posturing

10. __ None of the above

41. Is your child healthy, except for spitting up or vomiting?

1. ___ No

2. ___ Yes

42. For the past 3 months *when not vomiting*, did your child bring up food in his/her mouth after it has already been chewed and swallowed?

1. ___ No 🡪 *continue with question 48*

2. ___ Yes

43. At what age did the problem start?

1. ___ Birth

2. ___ 1 – 2 months

3. ___ 3 – 8 months

4. ___ 9 – 12 months

5. ___ Older than 12 months

44. Does the problem occur when your child is asleep?

1. ___ No

2. ___ Yes

45. Does the problem occur when your child is interacting with family or friends?

1. ___ No

2. ___ Yes

46. Does your child seem distressed during spitting up?

1. ___ No

2. ___ Yes

47. Is the problem better with:

a. Formula changes? __No __Yes

b. Medicine? __No __Yes

48. Has your child experienced at least 2 periods of unremitting paroxysmal vomiting with or without retching lasting hours to days within a six month period?

1. __ No

2. __ Yes

49. a. Between episodes of vomiting does your child return to his/her usual state of health for weeks or months?

1. __ No

2. __ Yes

b. Are episodes separated by weeks to months?

1. __ No

2. __ Yes

**PART 3**

**Information about the family**

50.Mother’s age in years: ......................................................................

51. Where is your family originally from? (e.g. The Netherlands, Indonesia, Turkey, Italy, Israel, Suriname, etc.): ……………………………………………………………………

52. Mother’s years of education: …………………………………………………………………….

53. Mother's employment: .........................................................................

54. Mother’s marital age in years: .........................................................................

55. Marital status:

- - 1. Married …… a. living with partner ……

b. living separate from partner ……

c. widow ……

d. divorced ……

- - 1. Not married …… a. living with partner ……

b. living separate from partner ……

c. single parent ……

56. Father’s age in years: ......................................................................

57. Where is your family originally from? (e.g. The Netherlands, Indonesia, Turkey, Italy, Israel, Suriname, etc.): ……………………………………………………………………

58. Father’s years of education: ……………………………………………………………………..

59. Father's employment: ..........................................................................

60. Father’s marital age in years: ..........................................................................

61. Marital status:

1. Married …… a. living with partner ……

b. living separate from partner ……

c. widower ……

d. divorced ……

1. Not married …… a. living with partner ……

b. living separate from partner ……

c. single parent ……

62. Household income/year:

1. ___ Less than ￥73,500,-
2. ___ ￥73,500- ￥146,999,-
3. ___ ￥147,000-￥257,249,-
4. ___ ￥257,250-￥367,499,-
5. ___ ￥367,500-￥735,000,-
6. ___ Over ￥735,000,-

63. Does your income meet your essential needs?

1. ___ No

2. ___ Yes

64. Does your family have any loans (mortgage not included)?

1. ___ No
2. ___ Yes

65. How is the relationship with your partner and family members who are living in your house?

- 1. ___ Very good
  2. ___ Good
  3. ___ Bad
  4. ___ Very bad

66. Place of residence:

- 1. ___ Urban area
  2. ___ Rural area

67. a. Was your family forced to change their place of residence because of security reasons or other related reasons?

- 1. ___ No
  2. ___ Yes

b. If yes, in which year? …………….

68. In the last two months, have you been subject to verbal violence in your own house?

- 1. __ Never
  2. __ Once in two months
  3. __ Once a month
  4. __ More than once a month
  5. __ Once a week
  6. __ More than once a week
  7. __ Daily

69. In the last two months, have you been subject to physical violence in your own house?

1. __ Never

2. __ Once in two months

3. __ Once a month

4. __ More than once a month

5. __ Once a week

6. __ More than once a week

7. __ Daily

70. In the last two months, has your baby been subject to verbal violence in your own house?

- 1. __ Never
  2. __ Once in two months
  3. __ Once a month
  4. __ More than once a month
  5. __ Once a week
  6. __ More than once a week
  7. __ Daily

71. In the last two months, has your baby been subject to physical violence in your own house?

1. __ Never
2. __ Once in two months
3. __ Once a month
4. __ More than once a month
5. __ Once a week
6. __ More than once a week
7. __ Daily
