# Supplementary material for: Prevalence of functional gastrointestinal disorders in infants and young children in China
Source: BMC Pediatr. 2021 Mar 17;21:131. doi: 10.1186/s12887-021-02610-6 (PMC7968152; doi:10.1186/s12887-021-02610-6)
Supplement: Supplementary file 2 — Additional file 2: Table 2. Early life factors and infant feeding practices against FGIDs. Associations between early life factors and infant feeding practices against FGIDs. [file 12887_2021_2610_MOESM2_ESM.doc]

**Table 2:** Early life factors and infant feeding practices against FGIDs

|  | | **Infant Colic** | | **Infant Regurgitation** | | **Infant Dyschezia** | | **Functional Diarrhoea** | | **Functional Constipation** | |
| --- | --- | --- | --- | --- | --- | --- | --- | --- | --- | --- | --- |
|  |  | **OR**  **(95% CI)** | **p value** | **OR**  **(95% CI)** | **p value** | **OR**  **(95% CI)** | **p value** | **OR**  **(95% CI)** | **p value** | **OR**  **(95% CI)** | **p value** |
| **Gender** | Male | 0.99  (0.70-1.41) | 0.967 | 1.38  (1.04-1.82) | **0.024*** | 0.90  (0.47-1.71) | 0.743 | 1.21  (0.38-3.82) | 0.744 | 1.04  (0.556-1.95) | 0.893 |
|  | Female | 1.01  (0.71-1.44) | 0.967 | 0.73  (0.55-0.96) | **0.024*** | 1.12  (0.58-2.13) | 0.743 | 0.83  (0.26-2.60) | 0.744 | 0.96  (0.51-1.79) | 0.893 |
| **Gestational Age (weeks)** | <34 | 0.31  (0.00-25.4) | 0.602 | 26.1  (0.59->999) | 0.092 | >999  (<0.00->999) | 0.767 | <0.00  (<0.00->999) | 0.978 | <0.00  (<0.00->999) | 0.973 |
|  | 35 – 37 | 0.21  (0.01-3.44) | 0.274 | 1.28 (0.08-19.4) | 0.859 | 0.26  (<0.00->999) | 0.980 | >999  (<0.00->999) | 0.951 | >999  (<0.00->999) | 0.923 |
|  | 38 – 42 | 0.37  (0.03-5.33) | 0.468 | 1.12  (0.08-15.9) | 0.935 | 0.03  (<0.00->999) | 0.950 | >999  (<0.00->999) | 0.954 | >999  (<0.00->999) | 0.948 |
|  | >42 | 3.24  (0.04-266) | 0.602 | 0.02  (<0.00-118) | 0.391 | <0.00  (<0.00->999) | 0.811 | <0.00  (<0.00->999) | 0.978 | <0.00  (<0.00->999) | 0.969 |
| **Birth Weight (kg)** | <1.5 | >999  (<0.00->999) | 0.989 | >999  (<0.00->999) | 0.981 | <0.00  (<0.00->999) | 0.931 | 0.58  (<0.00->999) | 0.999 | <0.00  (<0.00->999) | 0.964 |
|  | 1.6 – 2.0 | <0.00  (<0.00->999) | 0.965 | 0.26  (0.01-7.46) | 0.435 | <0.00  (<0.00->999) | 0.972 | 0.07  (<0.00->999) | 0.997 | <0.00  (<0.00->999) | 0.995 |
|  | 2.1 – 2.5 | 7.92  (1.37-45.8) | **0.021*** | 0.11  (<0.00->999) | 0.964 | >999  (<0.00->999) | 0.983 | 3.03  (<0.00->999) | 0.996 | 50.9  (<0.00->999) | 0.992 |
|  | 2.6 – 3.0 | 1.42  (0.89-2.25) | 0.138 | 0.82  (0.56-1.20) | 0.295 | >999  (<0.00->999) | 0.914 | 2.20  (<0.00->999) | 0.997 | >999  (<0.00->999) | 0.978 |
|  | >3.0 | <0.00  (<0.00->999) | 0.974 | 0.01  (<0.00->999) | 0.990 | >999  (<0.00->999) | 0.912 | 3.92  (<0.00->999) | 0.995 | >999  (<0.00->999) | 0.983 |
| **Growth Curve** | Normal | 0.57  (0.16-2.05) | 0.390 | 0.27  (0.03-2.76) | 0.271 | >999  (<0.00->999) | 0.847 | 0.14  (0.01-1.94) | 0.143 | >999  (<0.00->999) | 0.863 |
|  | Over-weight | 0.77  (0.09-6.40) | 0.809 | 12.4  (0.06-2513) | 0.352 | <0.00  (<0.00->999) | 0.951 | 1.04  (0.03-40.6) | 0.985 | >999  (<0.00->999) | 0.926 |
|  | Under-weight | <0.00  (<0.00->999) | 0.860 | 0.12  (<0.00-23.2) | 0.429 | >999  (<0.00->999) | 0.793 | <0.00  (<0.00->999) | 0.936 | >999  (<0.00->999) | 0.836 |
|  | Stunting | 3.75  (0.72-19.6) | 0.118 | 0.65  (0.01-46.9) | 0.845 | >999  (<0.00->999) | 0.795 | <0.00  (<0.00->999) | 0.907 | <0.00  (<0.00->999) | 0.928 |
|  | Wasted | 1.75  (0.49-6.28) | 0.390 | 3.81  (0.02-834) | 0.627 | <0.00 (<0.00->999) | 0.865 | >999  (<0.00->999) | 0.524 | <0.00  (<0.00->999) | 0.943 |
| **Mode of Delivery** | Vaginal | <0.00  (<0.00->999) | 0.881 | >999  (<0.00->999) | 0.957 | 0.03  (<0.00-1.57) | 0.083 | >999  (<0.00->999) | 0.936 | 0.01  (<0.00-0.17) | **0.003**** |
|  | Elective C-section | <0.00  (<0.00->999) | 0.883 | >999  (<0.00->999) | 0.958 | 0.04  (<0.00-2.27) | 0.119 | >999  (<0.00->999) | 0.915 | 0.03  (0.00-1.07) | 0.055 |
|  | Emergency C-section | <0.00  (<0.00->999) | 0.878 | >999  (<0.00->999) | 0.958 | 0.13  (<0.00-35.2) | 0.478 | <0.00  (<0.00->999) | 0.900 | <0.00  (<0.00-1.41) | 0.066 |
|  | Forceps Delivery | >999  (<0.00->999) | 0.738 | <0.00  (<0.00->999) | 0.957 | >999  (0.23->999) | 0.094 | 0.01  (<0.00->999) | 0.989 | >999  (154->999) | **0.002**** |
| **Exclusively Breast-feeding Duration (months)** | <1 | 1.34  (0.43-4.19) | 0.617 | 0.71  (0.33-1.52) | 0.382 | 0.55  (0.01-59.7) | 0.804 | 255  (<0.00->999) | 0.992 | <0.00  (<0.00->999) | 0.747 |
|  | 1 – 2 | 2.02  (0.74-5.51) | 0.170 | 4.54  (2.33-8.84) | **<.0001***** | 0.17  (0.00-11.1) | 0.410 | 5.68  (<0.00->999) | 0.995 | 34.4  (<0.00->999) | 0.891 |
|  | 2 – 3 | 1.97  (0.75-5.18) | 0.168 | 9.27  (4.71-18.3) | **<.0001***** | 0.26  (0.00-42.6) | 0.606 | >999  (<0.00->999) | 0.955 | >999  (<0.00->999) | 0.567 |
|  | 3 – 4 | 1.63  (0.71-3.74) | 0.250 | 4.29  (2.43-7.58) | **<.0001***** | 0.79  (0.01-60.0) | 0.916 | 0.14  (<0.00->999) | 0.992 | >999  (<0.00->999) | 0.702 |
|  | 4 – 6 | 0.75  (0.24-2.34) | 0.617 | 0.01  (<0.00-0.09) | **<.0001***** | 50.2  (0.23->999) | 0.156 | <0.00  (<0.00->999) | 0.933 | 134  (<0.00->999) | 0.848 |
| **Age of Formula Feeding Initiation (months)** | 0 – 1 | 1.06  (0.58-1.95) | 0.851 | 2.42  (1.46-3.99) | **0.001**  ****** | >999  (<0.00->999) | 0.731 | 0.04  (<0.00->999) | 0.997 | >999  (<0.00->999) | 0.758 |
|  | 1 – 2 | 0.76  (0.19-3.02) | 0.699 | 0.96  (0.39-2.37) | 0.934 | >999  (<0.00->999) | 0.697 | <0.00  (<0.00->999) | 0.934 | 0.07  (<0.00->999) | 0.922 |
|  | 2 – 3 | 0.57  (0.12-2.78) | 0.487 | 0.11  (0.03-0.34) | **0.0002***** | <0.00  (<0.00->999) | 0.927 | <0.00  (<0.00->999) | 0.790 | <0.00  (<0.00->999) | 0.511 |
|  | 3 – 4 | 0.53  (0.07-4.21) | 0.545 | 0.20  (0.06-0.69) | **0.011*** | <0.00  (<0.00->999) | 0.933 | >999  (<0.00->999) | 0.948 | <0.00  (<0.00->999) | 0.768 |
|  | 4 – 5 | <0.00  (<0.00->999) | 0.952 | 2.97  (0.60-14.6) | 0.181 | <0.00  (<0.00->999) | 0.943 | >999  (<0.00->999) | 0.870 | <0.00  (<0.00->999) | 0.761 |
|  | 5 – 6 | <0.00  (<0.00->999) | 0.976 | 1.27  (0.35-4.61) | 0.714 | <0.00  (<0.00->999) | 0.931 | >999  (<0.00->999) | 0.884 | 0.01  (<0.00->999) | 0.839 |
|  | Never | 0.94  (0.51-1.74) | 0.851 | 5.59  (0.23-136) | 0.291 | >999  (<0.00->999) | 0.778 | >999  (<0.00->999) | 0.934 | 0.00  (<0.00->999) | 0.798 |

*: p<0.05, **: p<0.01, ***: p<0.001
