# Supplementary material for: Prevalence of functional gastrointestinal disorders in infants and young children in China
Source: BMC Pediatr. 2021 Mar 17;21:131. doi: 10.1186/s12887-021-02610-6 (PMC7968152; doi:10.1186/s12887-021-02610-6)
Supplement: Supplementary file 3 — Additional file 3: Table 3.Socio-demographiccharacteristics against FGIDs. Association between socio-demographiccharacteristics against FGIDs. [file 12887_2021_2610_MOESM3_ESM.doc]

**Table 3:** Socio-demographic characteristics against FGIDs

|  | | **Infant Colic** | | **Infant Regurgitation** | | **Infant Dyschezia** | | **Functional Diarrhoea** | | **Functional Constipation** | |
| --- | --- | --- | --- | --- | --- | --- | --- | --- | --- | --- | --- |
|  |  | **OR**  **(95% CI)** | **p value** | **OR**  **(95% CI)** | **p value** | **OR**  **(95% CI)** | **p value** | **OR**  **(95% CI)** | **p value** | **OR**  **(95% CI)** | **p value** |
| **Residence Area** | Urban | 1.00  (0.69-1.45) | 0.991 | 0.70  (0.52-0.93) | **0.014*** | 1.29  (0.64-2.62) | 0.480 | 0.51  (0.13-1.95) | 0.324 | 0.63  (0.31-1.27) | 0.196 |
|  | Rural | 1.00  (0.69-1.45) | 0.991 | 1.44  (1.08-1.93) | **0.014*** | 0.78  (0.38-1.57) | 0.480 | 1.97  (0.51-7.58) | 0.324 | 1.59  (0.79-3.22) | 0.196 |
| **Annual Household Income (¥)** | <73,500 | >999  (<0.00->999) | 0.941 | 3.16  (0.30-33.1) | 0.337 | <0.00  (<0.00->999) | 0.868 | <0.00  (<0.00->999) | 0.947 | <0.00  (<0.00->999) | 0.863 |
|  | 73,500 – 146,999 | 0.61  (0.19-1.90) | 0.391 | 2.14  (0.23-19.6) | 0.500 | >999  (<0.00->999) | 0.856 | >999  (<0.00->999) | 0.734 | >999  (<0.00->999) | 0.872 |
|  | 147,000 – 257,249 | 0.92  (0.30-2.81) | 0.881 | 2.21  (0.25-19.9) | 0.478 | >999  (<0.00->999) | 0.839 | >999  (<0.00->999) | 0.762 | >999  (<0.00->999) | 0.852 |
|  | 257,250 – 367,499 | 0.76  (0.24-2.43) | 0.645 | 3.88  (0.43-34.9) | 0.227 | >999  (<0.00->999) | 0.799 | >999  (<0.00->999) | 0.823 | >999  (<0.00->999) | 0.820 |
|  | 367,500 – 735,000 | 0.37  (0.09-1.56) | 0.176 | 1.51  (0.16-14.4) | 0.722 | >999  (<0.00->999) | 0.830 | <0.00  (<0.00->999) | 0.727 | >999  (<0.00->999) | 0.773 |
|  | >735,000 | <0.00  (<0.00->999) | 0.870 | 0.01  (<0.00-645) | 0.421 | <0.00  (<0.00->999) | 0.929 | 0.24  (<0.00->999) | 0.989 | <0.00  (<0.00->999) | 0.939 |
| **Family Size** | 1 child only | 0.77  (0.05-13.3) | 0.858 | 0.34  (0.04-3.08) | 0.341 | 0.49  (0.01-24.9) | 0.721 | 0.36  (0.03-4.25) | 0.418 | 5.59  (0.59-52.7) | 0.133 |
|  | 2 – 3 Children | 1.30  (0.08-22.3) | 0.858 | 2.90  (0.32-26.0) | 0.341 | 2.05  (0.04-104) | 0.721 | 2.77  (0.24-32.6) | 0.418 | 0.18  (0.02-1.69) | 0.133 |
|  | ≥4 Children | - | - | - | - | - | - | - | - | - | - |
| **Birth Order** | 1 | 0.97  (0.00-324) | 0.992 | 10.1  (0.11-920) | 0.315 | >999  (<0.00->999) | 0.848 | 397  (<0.00->999) | 0.864 | 668  (<0.00->999) | 0.933 |
|  | 2 | 0.36  (0.02-8.08) | 0.519 | 0.87  (0.07-10.2) | 0.909 | >999  (<0.00->999) | 0.866 | 14.4  (<0.00->999) | 0.939 | >999  (<0.00->999) | 0.911 |
|  | 3 | 2.87  (0.67-123) | 0.583 | 0.11  (0.01-2.54) | 0.171 | <0.00  (<0.00->999) | 0.856 | <0.00  (<0.00->999) | 0.901 | <0.00  (<0.00->999) | 0.922 |
| **Paternal Education Level** | Primary | <0.00  (<0.00->999) | 0.932 | 1.08 (0.06-19.3) | 0.956 | <0.00  (<0.00->999) | 0.972 | 0.00  (<0.00->999) | 0.986 | >999  (<0.00->999) | 0.778 |
|  | Secondary | 1.10  (0.58-2.08) | 0.768 | 1.02  (0.63-1.67) | 0.923 | 0.77  (0.25-2.31) | 0.636 | 1.32  (0.19-9.14) | 0.780 | 39.0  (<0.00->999) | 0.967 |
|  | High School | 0.68  (0.33-1.42) | 0.305 | 1.05  (0.61-1.82) | 0.856 | 1.61  (0.50-5.18) | 0.423 | 2.09  (0.36-12.2) | 0.413 | 925  (<0.00->999) | 0.938 |
|  | College | >999  (<0.00->999) | 0.846 | 0.46  (0.00-51.6) | 0.745 | <0.00  (<0.00->999) | 0.860 | 5.86  (<0.00->999) | 0.987 | 0.34  (<0.00->999) | 0.990 |
|  | Bachelors | 0.91  (0.50-1.65) | 0.750 | 0.99  (0.63-1.55) | 0.949 | 0.59  (0.21-1.62) | 0.302 | 1.65  (0.40-6.71) | 0.487 | 90.9  (<0.00->999) | 0.959 |
|  | Masters | 0.97  (0.34-2.76) | 0.948 | 0.92  (0.40-2.09) | 0.834 | 0.31  (0.05-2.24) | 0.248 | 1.84  (0.22-15.4) | 0.574 | >999  (<0.00->999) | 0.934 |
|  | PhD | <0.00  (<0.00->999) | 0.935 | 2.04  (0.17-24.8) | 0.577 | <0.00  (<0.00->999) | 0.955 | <0.00  (<0.00->999) | 0.905 | <0.00  (<0.00->999) | 0.930 |
| **Maternal Education Level** | Primary | 3.43  (0.17-68.4) | 0.419 | 8.81  (0.65-120) | 0.103 | <0.00  (<0.00->999) | 0.970 | <0.00  (<0.00->999) | 0.966 | >999  (<0.00->999) | 0.841 |
|  | Secondary | 1.12  (0.51-2.47) | 0.775 | 1.26  (0.73-2.18) | 0.415 | 0.82  (0.26-2.57) | 0.731 | 0.46  (0.09-2.48) | 0.367 | 158  (<0.00->999) | 0.962 |
|  | High School | 2.32  (1.00-5.42) | 0.051 | 1.41  (0.76-2.62) | 0.280 | 0.31  (0.07-1.41) | 0.129 | 0.25  (0.02-2.60) | 0.244 | 249  (<0.00->999) | 0.958 |
|  | College | 0.29  (0.02-5.80) | 0.419 | 0.11  (0.01-1.55) | 0.103 | >999  (<0.00->999) | 0.896 | >999  (<0.00->999) | 0.939 | >999  (<0.00->999) | 0.927 |
|  | Bachelors | 1.45  (0.66-3.21) | 0.355 | 1.35  (0.77-2.36) | 0.299 | 0.73  (0.23-2.34) | 0.590 | 1.06  (0.28-3.98) | 0.934 | 14.0  (<0.00->999) | 0.980 |
|  | Masters | 3.39  (1.02-11.3) | **0.047*** | 1.11  (0.39-3.13) | 0.845 | 2.72  (0.42-17.6) | 0.293 | 8.93  (1.07-74.6) | **0.043*** | 18.1  (<0.00->999) | 0.978 |
|  | PhD | <0.00  (<0.00->999) | 0.955 | <0.00  (<0.00->999) | 0.929 | <0.00  (<0.00->999) | 0.973 | <0.00  (<0.00->999) | 0.944 | <0.00  (<0.00->999) | 0.941 |

*: p<0.05, -: no OR (95% CI) and p value
