# Supplementary material for: Prevalence of functional gastrointestinal disorders in infants and young children in China
Source: BMC Pediatr. 2021 Mar 17;21:131. doi: 10.1186/s12887-021-02610-6 (PMC7968152; doi:10.1186/s12887-021-02610-6)
Supplement: Supplementary file 4 — Additional file 4: Table 4. Stressful life events against FGIDs. Association between stressful life events against FGIDs. [file 12887_2021_2610_MOESM4_ESM.doc]

**Table 4:** Stressful life events against FGIDs

|  | | **Infant Colic** | | **Infant Regurgitation** | | **Infant Dyschezia** | | **Functional Diarrhoea** | | **Functional Constipation** | |
| --- | --- | --- | --- | --- | --- | --- | --- | --- | --- | --- | --- |
|  |  | **OR**  **(95% CI)** | **p value** | **OR**  **(95% CI)** | **p value** | **OR**  **(95% CI)** | **p value** | **OR**  **(95% CI)** | **p value** | **OR**  **(95% CI)** | **p value** |
| **Income Meets Needs** | Yes | 0.66  (0.34-1.28) | 0.216 | 1.03  (0.57-1.87) | 0.927 | 1.59  (0.31-8.10) | 0.575 | 1.07  (0.06-20.2) | 0.964 | 1.07  (0.26-4.35) | 0.923 |
|  | No | 1.52  (0.78-2.97) | 0.216 | 0.97  (0.54-1.77) | 0.927 | 0.63  (0.12-3.19) | 0.575 | 0.93  (0.05-17.6) | 0.964 | 0.93  (0.23-3.79) | 0.923 |
| **Family Relationship** | Very Good | 0.44  (0.03-5.68) | 0.531 | 0.32  (0.02-4.29) | 0.389 | 152  (<0.00->999) | 0.945 | >999  (<0.00->999) | 0.962 | >999  (<0.00->999) | 0.980 |
|  | Good | 0.48  (0.04-6.26) | 0.576 | 0.74  (0.05-10.1) | 0.818 | 476  (<0.00->999) | 0.932 | >999  (<0.00->999) | 0.961 | >999  (<0.00->999) | 0.978 |
|  | Bad | 2.27  (0.18-29.1) | 0.531 | 4.25  (0.03-723) | 0.581 | <0.00  (<0.00->999) | 0.938 | <0.00  (<0.00->999) | 0.962 | <0.00  (<0.00->999) | 0.979 |
|  | Very Bad | - | - | - | - | - | - | - | - | - | - |
| **Verbal Violence on Interviewee (mother / caregiver)** | Never | >999  (<0.00->999) | 0.978 | >999  (<0.00->999) | 0.968 | 0.17  (<0.00->999) | 0.991 | 0.02  (<0.00->999) | 0.998 | >999  (<0.00->999) | 0.991 |
|  | Once in 2 months | <0.00  (<0.00->999) | 0.978 | <0.00  (<0.00->999) | 0.968 | 5.87  (<0.00->999) | 0.991 | >999  (<0.00->999) | 0.994 | >999  (<0.00->999) | 0.981 |
|  | Once a month | - | - | - | - | - | - | 0.00  (<0.00->999) | 0.998 | <0.00  (<0.00->999) | 0.986 |
|  | >Once a month | - | - | - | - | - | - | - | - | - | - |
|  | Once a week | - | - | - | - | - | - | - | - | - | - |
|  | >Once a week | - | - | - | - | - | - | - | - | - | - |
|  | Daily | - | - | - | - | - | - | - | - | - | - |
| **Physical Violence on Interviewee (mother / caregiver)** | Never | >999  (<0.00->999) | 0.978 | <0.00  (<0.00->999) | 0.958 | >999  (<0.00->999) | 0.967 | 0.46  (<0.00->999) | 0.999 | 456  (<0.00->999) | 0.960 |
|  | Once in 2 months | <0.00  (<0.00->999) | 0.978 | >999  (<0.00->999) | 0.958 | <0.00  (<0.00->999) | 0.967 | 2.19  (<0.00->999) | 0.999 | 0.00  (<0.00->999) | 0.960 |
|  | Once a month | - | - | - | - | - | - | - | - | - | - |
|  | >Once a month | - | - | - | - | - | - | - | - | - | - |
|  | Once a week | - | - | - | - | - | - | - | - | - | - |
|  | >Once a week | - | - | - | - | - | - | - | - | - | - |
|  | Daily | - | - | - | - | - | - | - | - | - | - |
| **Verbal Violence on Subject (Infant / young child)** | Never |  | - | >999  (<0.00->999) | 0.989 |  | - | >999  (<0.00->999) | 0.920 | >999  (<0.00->999) | 0.926 |
|  | Once in 2 months | - | - | >999  (<0.00->999) | 0.991 | - | - | <0.00  (<0.00->999) | 0.952 | >999  (<0.00->999) | 0.959 |
|  | Once a month | - | - | <0.00  (<0.00->999) | 0.982 | - | - | <0.00  (<0.00->999) | 0.982 | >999  (<0.00->999) | 0.963 |
|  | >Once a month | - | - | - | - | - | - | 6.50  (<0.00->999) | 0.994 | <0.00  (<0.00->999) | 0.949 |
|  | Once a week | - | - | - | - | - | - | - | - | - | - |
|  | >Once a week | - | - | - | - | - | - | - | - | - | - |
|  | Daily | - | - | - | - | - | - | - | - |  | - |
| **Physical Violence on Subject (Infant / young child)** | Never | - | - | >999  (<0.00->999) | 0.986 | - | - | 20.4  (0-3.9E+163) | 0.987 | 0.08  (<0.00->999) | 0.456 |
|  | Once in 2 months | - | - | - | - | - | - | 0.00  (0-5.7E+134) | 0.970 | 6.51  (<0.00->999) | 0.484 |
|  | Once a month | - | - | <0.00  (<0.00->999) | 0.986 | - | - | <0.00  (<0.00->999) | 0.987 | >999  (<0.00->999) | 0.456 |
|  | >Once a month | - | - | - | - | - | - | - | - | - | - |
|  | Once a week | - | - | - | - | - | - | - | - | - | - |
|  | >Once a week | - | - | - | - | - | - | - | - | - | - |
|  | Daily | - | - | - | - | - | - | - | - | - | - |

-: no OR (95% CI) and p value
